# The Association between Triglyceride/High-Density Lipoprotein Cholesterol Ratio and All-Cause Mortality in Acute Coronary Syndrome after Coronary Revascularization

**DOI:** 10.1371/journal.pone.0123521

**Published:** 2015-04-16

**Authors:** Ke Wan, Jianxun Zhao, Hao Huang, Qing Zhang, Xi Chen, Zhi Zeng, Li Zhang, Yucheng Chen

**Affiliations:** 1 Department of Cardiology, West China Hospital, Sichuan University, Chengdu, Sichuan Province, P. R. China; 2 Division of Cardiology, the People’s Hospital of Sichuan Province, Chengdu, Sichuan, China; Heart Research Institute, AUSTRALIA

## Abstract

**Aims:**

High triglycerides (TG) and low high-density lipoprotein cholesterol (HDL-C) are cardiovascular risk factors. A positive correlation between elevated TG/HDL-C ratio and all-cause mortality and cardiovascular events exists in women. However, utility of TG to HDL-C ratio for prediction is unknown among acute coronary syndrome (ACS).

**Methods:**

Fasting lipid profiles, detailed demographic data, and clinical data were obtained at baseline from 416 patients with ACS after coronary revascularization. Subjects were stratified into three levels of TG/HDL-C. We constructed multivariate Cox-proportional hazard models for all-cause mortality over a median follow-up of 3 years using log TG to HDL-C ratio as a predictor variable and analyzing traditional cardiovascular risk factors. We constructed a logistic regression model for major adverse cardiovascular events (MACEs) to prove that the TG/HDL-C ratio is a risk factor.

**Results:**

The subject’s mean age was 64 ± 11 years; 54.5% were hypertensive, 21.8% diabetic, and 61.0% current or prior smokers. TG/HDL-C ratio ranged from 0.27 to 14.33. During the follow-up period, there were 43 deaths. In multivariate Cox models after adjusting for age, smoking, hypertension, diabetes, and severity of angiographic coronary disease, patients in the highest tertile of ACS had a 5.32-fold increased risk of mortality compared with the lowest tertile. After adjusting for conventional coronary heart disease risk factors by the logistic regression model, the TG/HDL-C ratio was associated with MACEs.

**Conclusion:**

The TG to HDL-C ratio is a powerful independent predictor of all-cause mortality and is a risk factor of cardiovascular events.

## Introduction

Cardiovascular disease (CVD) is the leading cause of mortality worldwide, and the majority of all CVD-related deaths occur in low- and middle-income countries [1]. A previous study has shown that severe hypertriglyceridemia is positively correlated with the mortality of CVD [2]. Atherogenic dyslipidemia (AD) is a major component of the metabolic syndrome and a strong predictor of coronary heart disease (CHD) [3]. Low-density lipoprotein (LDL) is a key factor in the pathogenesis of CHD. Decreasing the level of LDL-C is the primary goal of cardiovascular risk reduction therapy, and LDL particle—lowering agents, such as statins, significantly reduce CHD risk [4, 5]. Interestingly, the high triglycerides (TG) and low high-density lipoprotein cholesterol (HDL-C) ratio has been correlated strongly with the LDL particle size [6, 7]. The ratio of TG/HDL-C has been proposed to be an easily obtainable atherogenic marker.

Gaziano et al. was the first to identify that the TG/HDL ratio was a powerful predictor of myocardial infarction [8]. Hadaegh et al. suggested that the evaluation of this ratio should be considered for CHD risk prediction. [9] In women who are suspected to have myocardial ischemia, the ratio of TG to HDL-C is a strong independent predictor of all-cause mortality and cardiovascular death [10]. However, the outcomes of these related studies fail to assess the prognostic utility of the ratio of TG to HDL-C in acute coronary syndrome (ACS). Therefore, the goal of this study was to determine whether the ratio of TG/HDL-C predicts cardiovascular events and total mortality among ACS patients.

## Methods

### Study population

**Fig 1:**
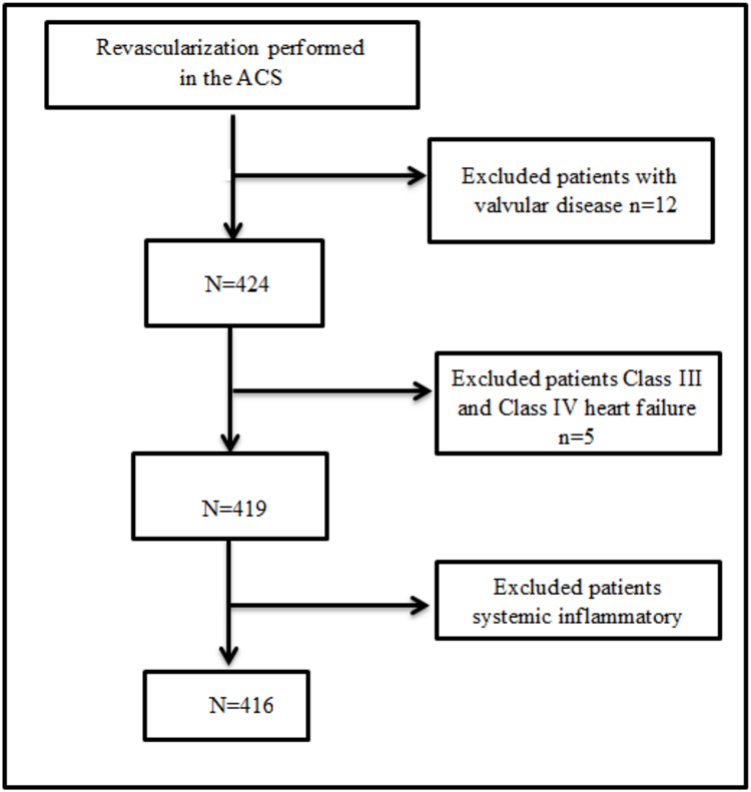
Flow chart of eligibility and exclusion of patients.

Discontinuous patients between 2006 and 2010 who were admitted to West China Hospital, were at least 18 years of age, had ACS, and were undergoing angiography were enrolled in this study. For the purposes of this study, patients with ACS were eligible for inclusion if they exhibited the following criteria: (1) stenosis of at least 50% in at least one proximal epicardial coronary artery; (2) ischemic chest discomfort that increased or occurred at rest and (3) elevated cardiac troponin T levels (≥0.03 mg/L); or new or presumably new electrocardiographic deviation in at least two contiguous leads [either pathologic Q waves (≥0.04 s in duration), ST segment dynamic horizontal/down-sloping depression ≥0.05 mV, or persistent ST segment elevation ≥0.1 mV in ≥2 contiguous precordial leads or ≥2 adjacent limb leads or new LBBB]. All patients were under revascularization. Major exclusion criteria included New York Heart Association Class III and Class IV heart failure, ongoing systemic inflammatory diseases, renal or hepatic dysfunction, significant valvular disease, myocarditis, cardiomyopathies, and malignancy. The flow diagram of the study is shown in the Fig 1. Twenty patients met the exclusion criteria and were dropped from the study; thus, the study population comprised 416 patients. The study was approved by the Ethics Committee of West China Hospital of Sichuan University. All subjects provided written informed consent before enrollment.

### Data collection

All participants completed a self-administered questionnaire that documented their medical history, current medications, and smoking habits (current smoker or not). The height and weight of participants were measured, and their body mass index (BMI) was calculated (kg/m2). For these measurements, participants wore light clothing without shoes. Blood pressures were measured and blood as well as urine sampling was done at each participant’s local medical institute, as stipulated by the health check program. Hypertension was defined as SBP ≥ 140 mm Hg or DBP ≥ 90 mm Hg, or if the patient was taking antihypertensive medication. DM was diagnosed according to one of the following criteria: (1) a history of diagnosed DM and treatment with medications, diet, and/or exercise; (2) non-fasting blood glucose of >11.1 mmol/L; (3) current use of hypoglycemic therapy to control DM.

### Laboratory methodology

A blood sample was drawn at 6:00 a.m. from all patients after an overnight fast to measure total cholesterol, LDL-C, HDL-C, and TG at the laboratory of the West China Hospital (Chengdu, China). TG/HDL-C was calculated as TG (mmol/L) divided by HDL-C (mmol/L). The patients were separately grouped into tertile based on their TG/HDL-C levels. TG/HDL-C levels for the tertile groups were as follows: lower 0.27–1.129, middle 1.129–2.067, upper 2.067 to -14.33.

### Coronary angiography

The severity of coronary atherosclerosis was evaluated according to the Gensini scoring system, which is used to reflect the severity of coronary artery disease (plaque rupture and progression of arteriosclerosis) by calculating the score based on the number of stenotic coronary artery segments, the degree of their lumen stenosis, and the localization of stenotic changes. Scoring is as follows: 1 for 1–25% narrowing, 2 for 26–50% narrowing, 4 for 51–75%, 8 for 76–90%, 16 for 91–99% and 32 for a completely occluded artery. This score was multiplied by a factor according to the importance of the coronary artery [11].

### Follow-up procedures

The primary outcomes of this study were death of any cause. The secondary outcomes were MACEs, assessed individually and as a composite, nonfatal myocardial infarction, including acute myocardial infarction (AMI) and old myocardial infarction (OMI), cardiac death, and revascularization. AMI was diagnosed in patients with symptoms of myocardial ischemia accompanied by an increase in a marker of myocardial necrosis. OMI was diagnosed in patients who exhibited no subjective symptoms at the time when an abnormal Q-wave was observed on the electrocardiogram. Cardiac death was documented as death related to MI, congestive heart failure, sudden cardiac death, or arrhythmia. Revascularization included any percutaneous coronary intervention (PCI) or coronary artery bypass grafting (CABG). After enrollment, care was provided by each patient's referring physician in accordance with local standards of care. Follow-up information was collected through contact with the patient’s physicians, the patients, or their family. All data were corroborated with the hospital records. In the event of death, a death certificate was obtained.

### Statistical analysis

For descriptive purposes, we compared means (SDs) or percentages, as appropriate, of demographic characteristics, cardiovascular risk factors, lipids and lipoproteins, medication use, angiographic coronary artery disease severity measures, and clinical outcomes across TG/HDL-C tertile. Because the distributions of TG/HDL-C and angiographic coronary artery disease severity score were skewed, these variables were log transformed before modeling. Covariates considered for the multivariable model included age, gender, history of hypertension, history of diabetes, history of smoking, and BMI. In separate models, we used Cox proportional hazard models to examine the association between TG/HDLC variables and mortality. The basic models adjusted for age and gender. We then sequentially added history of smoking (a stronger predictor in this cohort than current smoking), history of hypertension, and history of diabetes to determine whether the relationship between TG/HDL-C and clinical outcomes was independent of these covariates. The associations between the log of the TG/HDL-C ratio and MACEs, respectively, were modeled using multiple logistic regression analysis models. In addition, we considered the use of lipid-lowering medications, but use was not predictive of death or cardiovascular events and did not affect the relationship between log TG/ HDL-C and outcomes. Thus, BMI and use of lipid-lowering medications were not included in the models. The coronary artery severity score (the strongest predictor among the angiographic coronary artery disease severity measures) was then added to the models. The proportional hazards assumption of invariant hazard ratios during follow-up was tested and found to be met. P-values of 0.05 or less were considered statistically significant. All analyses were conducted using SPSS software, version 17.0, and all tests for statistical significance were 3 tailed. The authors are solely responsible for study design and conduct as well as all study analyses, and the drafting and editing of this paper and its final contents.

## Results

### Baseline characteristics

**Fig 2:**
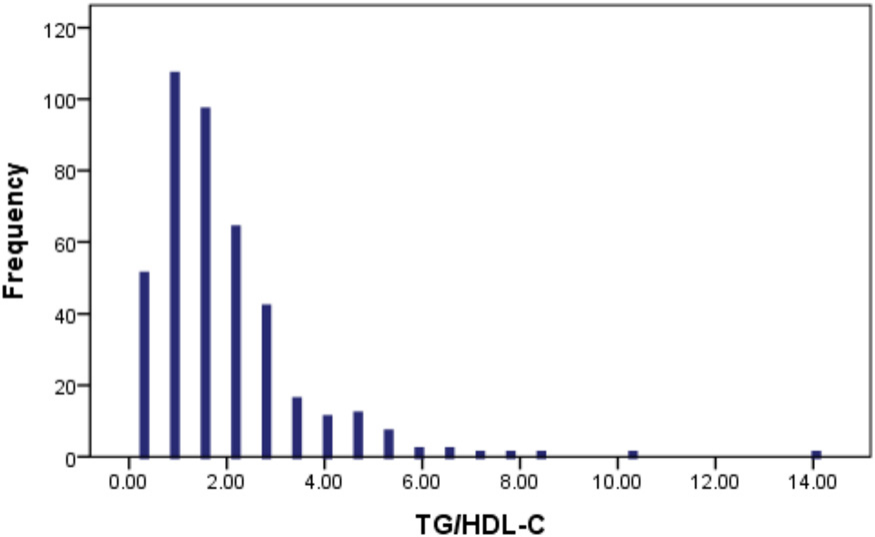
Distribution of the TG/HDL-C ratio in the study population.

Baseline characteristics of the 416 patients in the study cohort are shown in Table 1. The average age was 64 ± 11 years, 54.5% had a history of hypertension, 21.8% were diabetic, and 61.0% had a history of smoking. Table 1 summarizes baseline characteristics by TG/HDL-C tertiles. Age was similar across tertile. ACS patients with higher TG/HDL-C ratio were likely to be yanger. Smoking rates varied by group, but there was no consistent trend with increasing TG/HDL-C ratio. Among ACS patients, there were no different components of the metabolic syndrome; none fulfilled the criteria for diabetes or CAD severity score. Use of medications known to affect cardiovascular outcomes (aspirin, lipid-lowering drugs, β blockers, angiotensin-converting enzyme inhibitors or angiotensin receptor blockers, and hormone replacement therapy) did not differ across TG/HDL-C strata. The mean TG/HDL-C ratio with TG and HDL-C expressed in millimoles per litre was 1.91 ± 1.51; the median was 1.54 and range was 0.33 to 14.33 (Fig 2).

**Table 1:** Baseline characteristics according to the TG/HDL-C ratio.

Characteristic	Lower (n = 139)	Middle (n = 138)	Upper (n = 139)	P-value
Age (years)	68.32±8.64	63.36±11.34	62.76±11.80	<0.001
Male (%)	74.8	73.2	77.0	0.766
History of smoking (%)	56.8	59.4	66.9	0.202
BMI	23.41±3.35	24.37±3.71	23.99±2.96	0.271
Diabetes (%)	19.4	23.2	23.0	0.693
Hypertension (%)	52.5	56.5	44.6	0.13
**Lipid measures**				
TC(mmol/L)	3.78±0.91	3.87±1.58	3.64±1.74	0.404
HDL-C(mmol/L)	1.57±0.78	1.33±0.83	1.09±0.49	<0.001
LCL-C(mmol/L)	2.14±0.77	2.55±1.22	2.59±1.00	<0.001
TG(mmol/L)	1.17±0.70	2.05±1.30	3.60±1.90	<0.001
TG/HDL-C	0.73±0.23	1.56±0.26	3.39±1.67	<0.001
**CAD severity score**				
Gensini score	32.0 (12.5–65.0)	28.0 (14.0–60.5)	15.0 (32.8–64.4)	0.814
**Medications**				
Aspirin (%)	73	69.7	79.5	0.214
Statins (%)	62.3	68.0	76.1	0.07
β blockers (%)	60.7	65.6	70.9	0.246
Clopidogrel (%)	32.8	36.1	41.0	0.413
ACEI (%)	14.8	18.0	16.2	0.786
ARB (%)	27.9	33.6	36.8	0.331

CAD, Coronary artery disease; ACE-I, angiotensin-converting enzyme inhibitor; ARB, angiotensin receptor blocker. The ratio of TG/HDL-C is expressed with TG and HDL-C in millimoles per litre.

### Survival

Mean follow-up time for ACS was 33.3 ± 0.40 months. When the events were analyzed with regard to the TG/HDL-C distribution, the deaths at the end of the follow-up period were 10 and 22 in the middle and upper tertile, respectively. 11 deaths occurred in the lower tertile.

**Fig 3:**
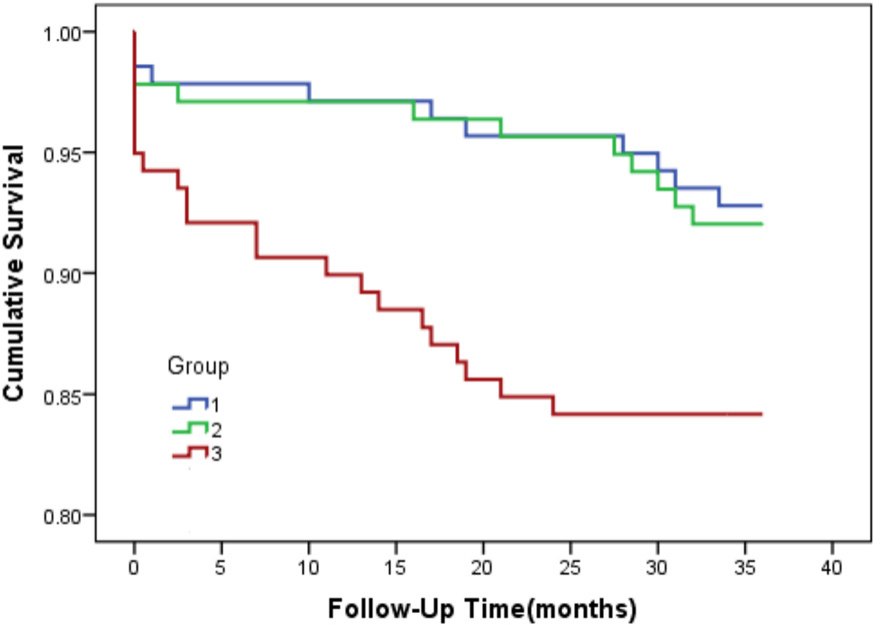
Kaplan-Meier curves depicting freedom from all-cause mortality according to the TG/HDL-C tertile.

All-cause mortality is significantly more common in ACS patients with higher TG/HDL-C tertiles. Kaplan-Meier curves for freedom from death events are shown in Fig 3 for the TG/HDLC ratio tertiles. Survival free of all-cause mortality decreased with increasing tertiles of TG/HDL-C ratio. After adjusting all confounding factors, CAD patients with the highest tertile of LDL/HDL-C ratio exhibited multivariable-adjusted HRs of 5.32 (95% CI 2.06–13.73) for all-cause mortality compared with CAD patients with the lowest quartile of LDL/HDL-C ratio. Results of the sequential modeling of all-cause mortality are shown in Table 2. When logarithmically transformed, the ratio of continuous variables was consistent with categorical variables (Table 3). The log TG/HDL-C was a powerful predictor of death events independent of age, gender, smoking, and diabetes, and remained predictive after the coronary artery disease severity score was added to the model.

**Table 2:** HRs for all-cause mortality according to TG/HDL-C ratio.

TC/HDL ratio	Model 1	Model 2	Model 3	Model 4
Lower	1	1	1	1
Middle	1.44 (0.61–3.40)	1.44(0.61–3.41)	1.33(0.56–3.15)	1.15(0.47–2.86)
Upper	3.15(1.49–6.69)	3.107(1.46–6.62)	3.02(1.41–6.48)	5.32(2.06–13.73)
P-value	0.002	0.002	0.003	0.000

Model 1 was adjusted for age, gender. Model 2 was adjusted for age, gender, smoking. Model 3 was adjusted for age, gender, smoking, history of hypertension and history of diabetes. Model 4 was adjusted for age, gender, smoking, history of hypertension, history of diabetes, and CAD severity score.

**Table 3:** Cumulative incidence of the composite endpoint of all-cause death by TG/HDL-C.

Predictor	Model 1		Model 2		Model 3		Model 4	
	HR (95% CI)	P	HR (95% CI)	P	HR (95% CI)	P	HR (95% CI)	p
Log TG/HDL	8.84(3.14–24.89)	0.000	8.83(3.09–25.21)	0.001	7.98(2.78–22.90)	0.000	7.79(2.51–24.20)	0.001
Age(years)	1.07(1.04–1.11)	0.000	1.08(1.04–1.12)	0.000	1.07(1.03–1.11)	0.000	0.97(0.92–1.02)	0.210
Gender(male)	1.03(0.53–2.00)	0.942	0.60(0.27–1.35)	0.128	0.92(0.40–2.10)	0.837	0.79(0.40–1.59)	0.511
Smoking			2.47(1.10–5.60)	0.006	1.89(0.85–4.22)	0.119	0.56(0.27–1.20)	0.136
Hypertension					2.25(1.08–4.67)	0.030	1.58(0.75–3.33)	0.228
Diabetes					1.99(1.06–3.77)	0.034	1.06(0.57–1.96)	0.864
Log Gensini							2.79(0.92–8.37)	0.069

### Cardiovascular events

There were 75 cardiovascular events. To investigate the potential independent contribution of the TG/HDL-C ratio on ACS, a multiple logistic regression analysis was performed. After adjusting for other conventional risk factors of CAD, including age, gender, smoking, hypertension, and diabetes, the TG/HDL-C ratio was associated with cardiovascular mortality [the adjusted P-value was 0.016 and OR was 2.94 (95%CI 1.22–7.09); Table 4].

**Table 4:** Multivariable analysis of the association between TG/HDL-C ratio and MACEs.

	crude		Adjust	
	OR(95% CI)	P	OR(95% CI)	P
Log TG/HDL	2.36(1.02–5.49)	0.046	2.94(1.22–7.09)	0.016

Adjusted for age, gender, hypertension, smoking, diabetes by using the logistic regression model.

## Discussion

To our knowledge, this is the first study on the prognostic utility of the TG/HDL-C ratio among ACS patients. The main finding of this study is that the TG/HDL-C ratio is a powerful independent predictor of total mortality of important prognostic variables, including age, smoking, hypertension, and diabetes. In addition, we found that the TG/HDL-C ratio is a risk factor for subsequent cardiovascular disease after coronary revascularization.

The TG concentration was a negative independent variable and the HDL-C concentration was a positive independent variable predicting LDL size, and the TG/HDL-C ratio was beneficial for assessing the presence of small LDL [12]. A low ratio of TG/HDL-C was represented primarily by large, non-atherogenic LDL particles; however, a high ratio of TG/HDL-C represented a larger group of small, dense pro-atherogenic LDL particles, which was strongly correlated to the initiation and progression of atherosclerosis [13]. Dobiasova et al. found that the plasma parameter log of TG/HDL-C as an atherogenic index represents the higher plasma concentration of triglyceride-rich, very low-density lipoproteins that generate small, dense LDL during lipid exchange and lipolysis [13].

Based on the findings of previous studies, we deduced that there was a positive correlation between the TG/HDL-C ratio and vascular change and damage, and the ratio of TG/HDL-C was a useful predictor for CHD. The TG to HDL-C ratio has been firmly believed to be an accurate marker of the independent risk factors for vascular changes, insulin resistance (IR) [14, 15], metabolic syndrome(MetS) [16], and hypertension [17]. The TG/HDL-C ratio and IR in severely obese non-diabetic individuals was positively correlated [18–20]. Bittner et al. stated that the TG/HDL-C ratio was an independent predictor of all-cause mortality in those females who have suspected myocardial ischemia [10].

In addition, there have been a few studies on the relationship between the TG to HDL-C ratio and vascular damage [21–24]. In a study examining the associations between CHD risk markers and carotid intima-media thickness (cIMT) progression in subjects at moderate risk of CHD, the ratio of TG to HDL-C independently predicted cIMT progression [25]. Shimizu et al. found that there was a significant positive correlation between diabetes, especially associated with high TG/HDL-C ratio and an evaluated risk of cIMT and arterial stiffness [23]. TG/HDL-C and HDL-C have been demonstrated by a receiver operating characteristic curve analysis to be a useful marker for the detection of the extent of coronary disease. [26]. Gaziano et al. showed that the log TG/HDL-C ratio predicted the risk for myocardial infarction [3]. Even after adjusting for non-lipid risk factor, LDL peak particle size and LDL-C concentrations, log TG/HDL-C ratio was found to be independently predictive of the risk of ischemic heart disease [27]. Furthermore, studies have shown that the TG/HDL-C ratio is strongly associated with the severity of coronary disease [26]. The higher values of the TG/HDL-C ratio has been associated with the higher risk of cardiovascular events even though LDL-C is low or lowered by active remedy with non-reduction in the TG to HDL-C ratio [28, 29].

As in prior studies, the current study found that the TG/HDL-C ratio was an independent predictor of cardiovascular events and all-cause mortality in ACS after coronary revascularization, with about five-fold increase in three-year event rates in the highest tertile of the TG/HDL-C distribution compared with the tertile quartile of the TG/HDL-C distribution. We discovered, after adjusting for demographic variables and traditional coronary risk factors, including diabetes, that TG/HDL-C remained a predictor of cardiovascular events.

As the Kaplan-Meier curves show (Fig 3), this increased risk seems to be confined to the highest tertile of the TG/HDL-C distribution, with a TG/HDL-C ratio of ≥2.067. The ratio when transformed by the continuous variables in the Cox proportional hazard models, the TG/HDL hazard ratio was decreased by further adjusting for the severity of ACS. This suggests that TG/HDL-C’s covariation with the severity of disease might explain some of the high TG/HDL-C associated risks. The decrease of the TG/HDL-C hazard ratio may also indicate ACS to be the more proximal of the 2 variables to adverse events suggesting a possible causal pathway. Such a pathway would assume the TG/HDL-C ratio to be relatively consistent over time.

In conclusion, even after adjusting for traditional risk factors and the severity score of ACS, the TG/HDL-C ratio remained to be the independent predictor of all-cause mortality in our subjects. Due to the absence of the cause of death information and our study design, we cannot assess the pathophysiological mechanisms that underlie the strong relationship between the TG/HDL-C ratio and subsequent all-cause mortality among patients with ACS. However, our data suggest that ACS patients with a high TG/HDL-C ratio should be considered high risk of death and should be closely followed-up clinically.
